# Supplementary figures and images for: Plasmodium-encoded murine IL-6 impairs liver stage infection and elicits long-lasting sterilizing immunity
Source: Front Immunol. 2023 Apr 11;14:1143012. doi: 10.3389/fimmu.2023.1143012 (PMC10152192; doi:10.3389/fimmu.2023.1143012)

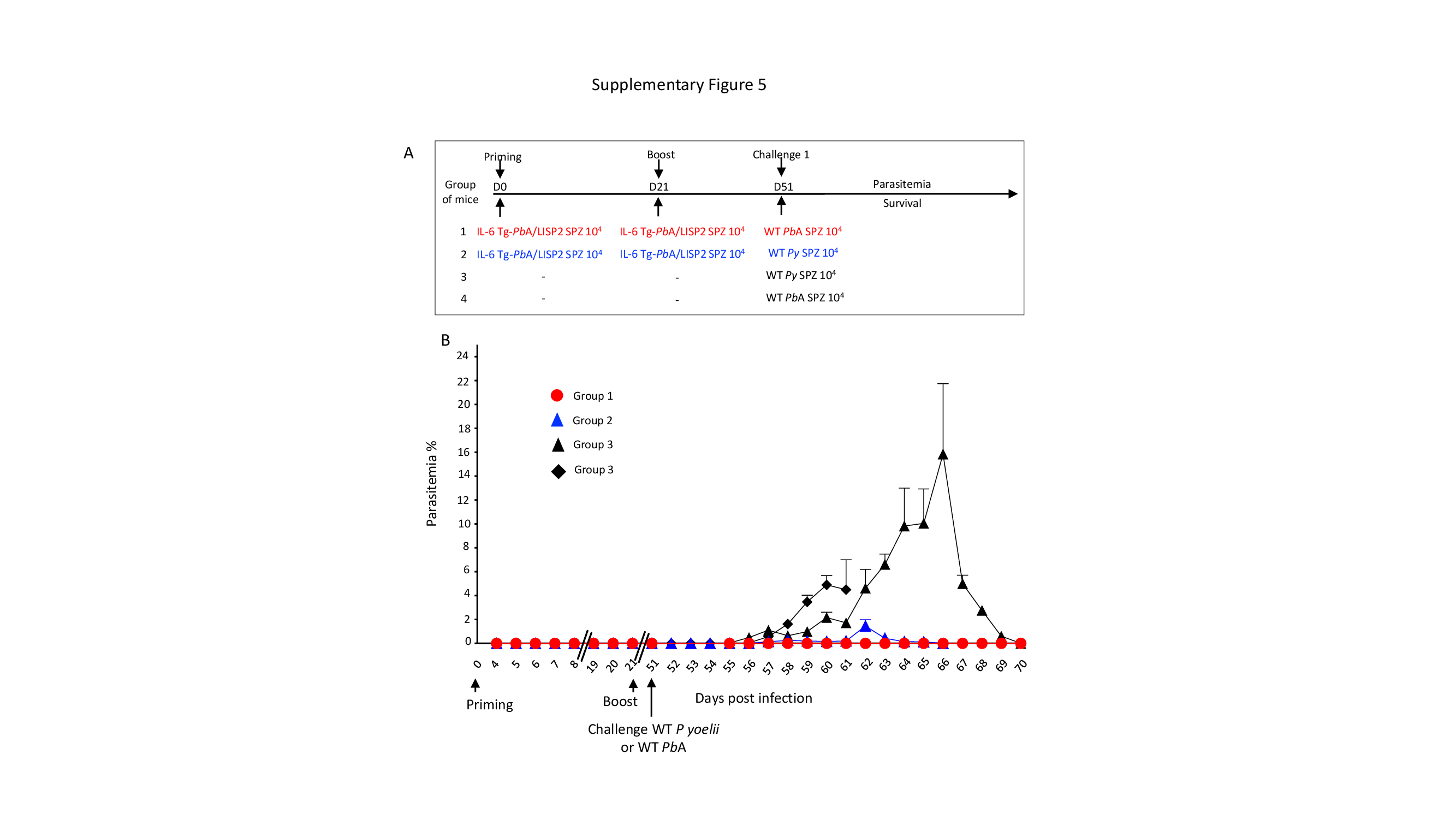

Supplement: Supplementary Figure 5 — Prime/boost immunization regimen with IL-6 Tg-PbANKA/LISP2 parasites confers a stable and efficient protection against challenge with heterologous P. yoelii 17XNL SPZ. (A) Groups of C57BL/6 mice were infected twice with 104 IL-6 Tg-PbA/LISP2 SPZ at 3 weeks interval, and then challenged 30 days later (day 51) with either 104 WT PbANKA SPZ (Group 1) or with WT P. yoelii 17XNL SPZ (group 2). Control groups of age-matched naïve mice received only 104 WT P. yoelii 17XNL SPZ (Group 3) or WT PbANKA SPZ (Group 4). (B) Parasite development was measured at indicated time points by flow cytometry, as all parasites were tagged with GFP. Error bars, SEM. Data are representative of one experiment with 5 mice per group. [file Image_5.tif]

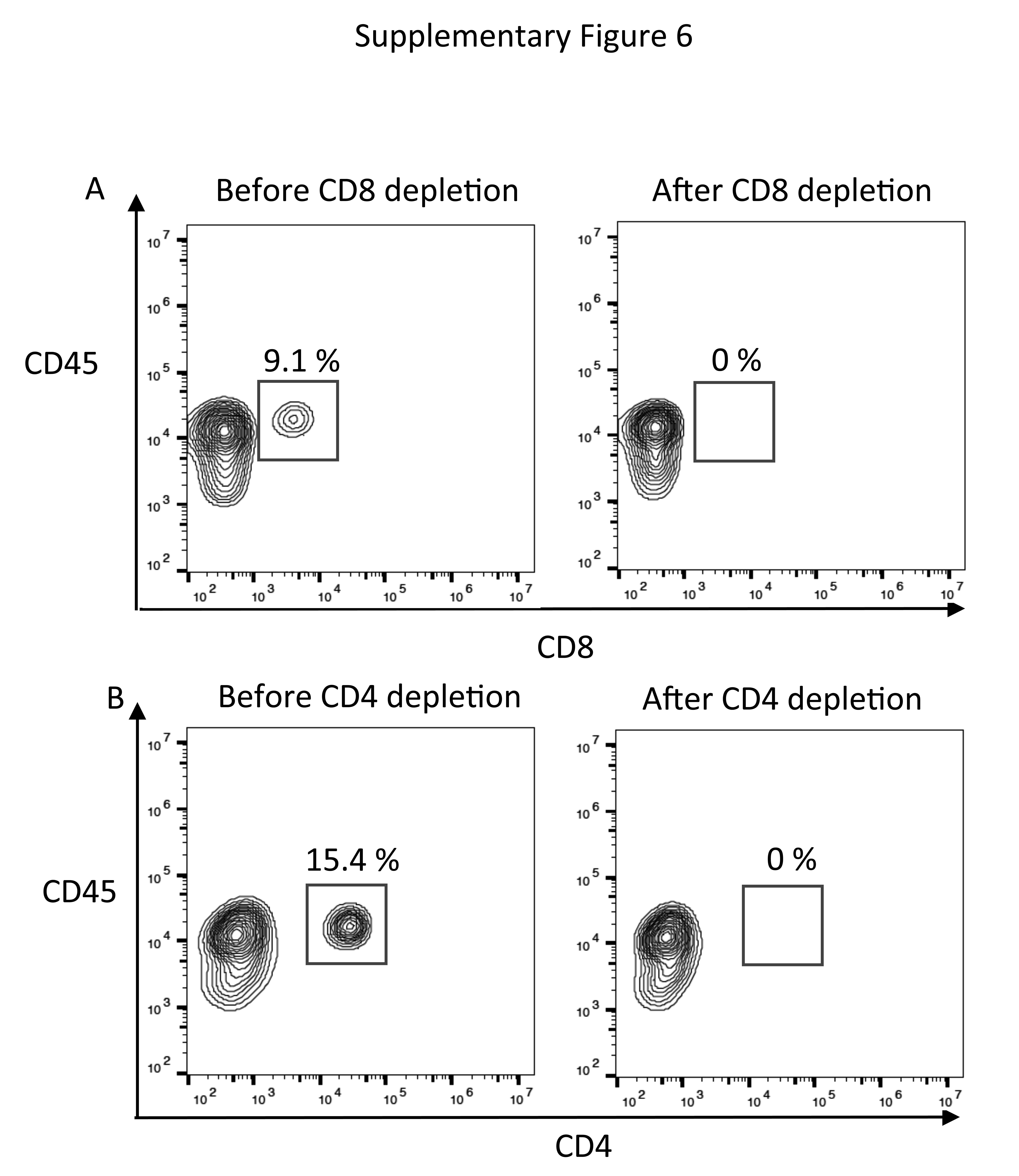

Supplement: Supplementary Figure 6 — Assessment of leukocyte depletion. In vivo depletion of CD4+ or CD8+ T cells in immunized mice using anti-CD4 or anti-CD8 depleting antibodies was assessed by measuring daily the percentage of residual (A) CD8+ or (B) CD4+ T cells in the blood by FACS analysis. Typical analysis performed at day 2 post treatment, corresponding to the day of challenge with WT PbANKA SPZ (refer to ), is shown in this figure. Representative data from two independent experiments with 5 mice per group are shown. [file Image_6.tif]
